# Use of allele scores as instrumental variables for Mendelian randomization

**DOI:** 10.1093/ije/dyt093

**Published:** 2013-08-30

**Authors:** Stephen Burgess, Simon G Thompson

**Affiliations:** Department of Public Health and Primary Care, Worts Causeway, Cambridge CB1 8RN, UK

**Keywords:** Mendelian randomization, allele scores, genetic risk scores, instrumental variables, weak instruments

## Abstract

**Background** An allele score is a single variable summarizing multiple genetic variants associated with a risk factor. It is calculated as the total number of risk factor-increasing alleles for an individual (unweighted score), or the sum of weights for each allele corresponding to estimated genetic effect sizes (weighted score). An allele score can be used in a Mendelian randomization analysis to estimate the causal effect of the risk factor on an outcome.

**Methods** Data were simulated to investigate the use of allele scores in Mendelian randomization where conventional instrumental variable techniques using multiple genetic variants demonstrate ‘weak instrument’ bias. The robustness of estimates using the allele score to misspecification (for example non-linearity, effect modification) and to violations of the instrumental variable assumptions was assessed.

**Results** Causal estimates using a correctly specified allele score were unbiased with appropriate coverage levels. The estimates were generally robust to misspecification of the allele score, but not to instrumental variable violations, even if the majority of variants in the allele score were valid instruments. Using a weighted rather than an unweighted allele score increased power, but the increase was small when genetic variants had similar effect sizes. Naive use of the data under analysis to choose which variants to include in an allele score, or for deriving weights, resulted in substantial biases.

**Conclusions** Allele scores enable valid causal estimates with large numbers of genetic variants. The stringency of criteria for genetic variants in Mendelian randomization should be maintained for all variants in an allele score.

## Introduction

Allele scores (also called genetic risk scores, gene scores or genotype scores) are a convenient way of summarizing a large number of genetic variants associated with a risk factor. An unweighted allele score is constructed as the total number of risk factor-increasing alleles present in the genotype of an individual. A weighted allele score can also be considered, where each allele contributes a weight reflecting an estimate of the effect of the corresponding genetic variant on the risk factor. These weights can be internally derived from the data under analysis, or externally derived from prior knowledge or an independent data source. In this way, multidimensional genetic data on variants associated with a risk factor can be collapsed into a single variable. Allele scores have been constructed for many traits, including fasting,^1^ blood pressure^2^ and high-density lipoprotein cholesterol.^3^

Allele scores are important for the modelling of multifactorial polygenic traits, particularly when the allele score consists either of many common variants with small effects, or of rare variants. When several such variants are combined into an allele score, the score may explain a considerable proportion of variation in the risk factor, even if none of the variants individually does.

### Mendelian randomization

In this paper, we consider the use of allele scores in Mendelian randomization: that is the application of instrumental variable methods with genetic instruments to estimate the causal effect of a risk factor on an outcome from observational data.^4^^,^^5^ Under the assumption that the genetic instruments used are specifically associated with the risk factor of interest, and not directly associated with either the outcome or any potential confounding variable, a genetic instrumental variable divides the population into subgroups which systematically differ in the risk factor, but not in any competing risk factor.^6^ The genetically-defined subgroups are analogous to treatment arms in a randomized controlled trial.^7^ Any difference in the outcome between the subgroups is inferred to be causally due to the risk factor of interest, subject to the validity of the instrumental variable assumptions.^8^

In this paper, we assume the context of a continuous risk factor and a continuous outcome. In order to consistently estimate a causal effect, further structural assumptions are necessary, such as linearity in the association between the risk factor and the outcome. These assumptions have been discussed at length elsewhere,^6^^,^^9^ and are assumed to hold in this paper.

### Violation of instrumental variable assumptions

Violation of the instrumental variable assumptions can occur for a number of biologically plausible reasons, including pleiotropic association of the genetic variant with a confounding variable or with the outcome directly, linkage disequilibrium with another functional variant associated with a confounding variable or the outcome, and population stratification where genetic associations reflect latent strata in the population.^10^^,^^11^ However, where there is substantial scientific evidence on a genetic variant to justify its use as an instrumental variable, the instrumental variable estimate can be reasonably assumed to represent a causal effect. Examples of genetic variants which have been used in this way for coronary heart disease include variants in the *CRP* gene for the causal effect of C-reactive protein,^12^ and variants in the *IL6R* gene for the causal effect of interleukin-6 receptor.^13^

## Using allele scores in Mendelian randomization

Allele scores are used in Mendelian randomization for reasons of simplicity, increased power^14^ and avoidance of weak instrument bias.^15^ Their use requires the assumption that the allele score is an instrumental variable/cite,^16^ and so is specifically associated with the risk factor and not with the outcome or confounders as above. This means that each variant which contributes to the allele score must be an instrumental variable.^14^ As the biological effects of all the variants in an allele score may not be well known, the instrumental variable assumptions may not be satisfied for all the variants. We demonstrate the problems resulting from departures from these assumptions, as well as from assumptions which are commonly made for mathematical convenience, such as the use of additive genetic models with no interactions between genetic variants. The aim of this paper is to show how use of an allele score resolves some of these problems; first in an idealized setting, and then in a range of more realistic scenarios.

### Examples of allele scores used in practice

To motivate the methodological issues considered in this paper, we here provide some examples of how allele scores have been used in practice. Lin *et al.*^17^ used an unweighted and a weighted allele score based on 15 genetic variants in the context of risk prediction, deriving weights from the data under analysis. They found that a weighted allele score provided greater discrimination than an unweighted score when used in conjunction with conventional risk factors. Rasmussen *et al.*^1^ and Ehret *et al.*^2^ used a weighted allele score in the context of Mendelian randomization, deriving weights from the data under analysis. Rasmussen *et al.* chose five variants from genetic regions which showed significant *P*-values in the dataset, although the precise choice of variants was from a separate meta-analysis (which included the study under analysis). In Ehret *et al.*, several of the 29 variants used in the allele score were novel, and were chosen on the basis of *P*-values in the dataset. Voight *et al.*^3^ used a weighted allele score with 14 variants to perform Mendelian randomization, deriving weights from a published meta-analysis, although some studies were in common between the two analyses. It is not clear how the specific variants were chosen, although they are reported as having significant *P*-values in the dataset.

## Simulation study

In order to evaluate the performance of various methods for causal estimation with multiple genetic variants and allele scores, we undertake a simulation study. The study is presented as a ‘theme and variations’, with an initial analysis performed where each of the genetic variants is a valid instrumental variable for the risk factor and has the same magnitude of effect on the risk factor, and with further analyses varying the data-generating mechanism and comparing methods used for constructing the allele score and for instrumental variable analysis.

### Initial analysis: valid instruments with equal-sized effects

We generate simulated data for a risk factor (*X*) which is a linear sum of a confounder (*U*), assumed unmeasured, a set of *J* independently distributed genetic variants (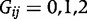 for *j* = 1 , … , J) representing the number of minor alleles for each variant and a normally distributed error term. The outcome (*Y*) is a continuous variable calculated as the linear sum of the risk factor, the confounder and an independent error term. The initial data-generating model for individual *i* is:
(1)
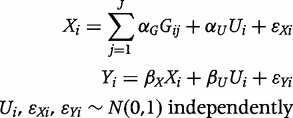



Data are simulated with 9, 25 and 100 genetic variants for 3000 individuals. We set 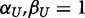 so that the risk factor and outcome are positively correlated even without a causal effect of the risk factor on the outcome. Three values are taken for the causal effect 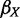 of 0, 0.2 and 0.4. We choose 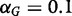 for 9 variants, 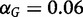 for 25 variants and 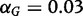 for 100 variants with a minor allele frequency of 0.3 for each variant, so that the proportion of variation in the risk factor explained by the genetic variants above that expected by chance (the adjusted 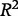) is approximately 1.9% throughout, similar to the 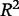 for the allele score. This is a fairly typical proportion for many biomarkers.^12^ Although many traits have a heritability which is much greater than 1.9%,^18^ it is unlikely that this heritability can be attributed to genetic variants which are specifically associated with the trait of interest rather than those associated with potential confounders.

For each of 1000 simulated datasets, we calculate estimates of the causal effect using an unweighted allele score (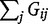) as an instrumental variable and the two-stage least squares (2SLS) method to give a point estimate and standard error.^19^ In comparison, we also present results using the 2SLS and limited information maximum likelihood (LIML) methods^20^ with a multivariable first-stage regression model for the genetic association with the risk factor using a single coefficient (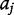) for each genetic variant:

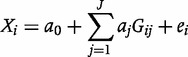



Analyses are implemented using the *ivreg2* command in Stata^21^ (LIML) and the *tsls* command^22^ in R^23^ (2SLS). LIML and 2SLS give identical estimates in the case of a single instrumental variable such as an allele score. We focus on the properties of bias, empirical coverage (proportion of datasets in which the 95% confidence interval contains the true causal effect), and empirical power (proportion of datasets detecting a non-null causal effect at a significance level of 5%).

When the strength of the instruments is low, estimates using multiple instruments are known to be biased in the direction of the observational confounded association and have non-normal distributions,^24^^,^^25^ leading to poor coverage properties in analysis methods relying on asymptotic standard errors.^26^ The strength of the instruments is measured by the F statistic from the regression of the risk factor on the instruments.^27^ Conventionally, instruments with an F statistic less than 10 are labelled as ‘weak’,^28^ although so-called ‘weak instrument bias’ is a continuous rather than a binary phenomenon. The bias is a result of over-fitting in the genetic model, whereby the genetic variants explain not only systematic variation in the risk factor of interest, but also chance variation in the confounders; the bias is towards the confounded observational estimate.^29^ Instruments with lower expected F statistics correspond to estimates which are more biased. A weak instrument should not be confused with an invalid instrument, and estimates using a weak instrument will be consistent for the causal effect with a large enough sample size.

Whereas the use of F statistic thresholds for controlling bias has been advocated by some,^30^ naive application of these rules can exacerbate bias rather than reduce it. We believe that thresholds are unhelpful to applied researchers in understanding weak instruments, as: (i) they encourage researchers to classify instruments into the binary classes of weak or non-weak, rather than acknowledging that weak instrument bias is a continuous phenomenon; (ii) they ignore the substantial sampling variation in the F statistic—the bias of the 2SLS estimator depends on the expected value of the F statistic, not the estimate of the F statistic in the given dataset; and (iii) they promote the selection of studies and instruments based on a data-derived statistic, leading to *post hoc* choice of analysis and substantial potential bias.^26^^,^^29^

In our simulations, the mean F statistic from the regression of the risk factor on the allele score is almost 60, meaning that causal estimates using the allele score should be unaffected by weak instrument bias. The F statistics using each of the genetic variants as a separate instrumental variable are much lower. Estimates from the LIML method are less affected by weak instrument bias than those from the 2SLS method.^31^

In further simulations, scenarios 1 to 7 below, we consider various departures from the data-generating model (1) which reflect practical issues relating to constructing and using allele scores. We examine how estimates from instrumental variable methods using an allele score are affected by these changes. Unless otherwise stated, all parameters take the same values as in the initial simulation. In the main paper, we describe situations with 25 variants; results from models with 9 and 100 variants obtained by scaling the genetic parameters accordingly are given in the Supplementary Appendix (available as Supplementary data at *IJE* online). Parameters are chosen to take plausible values with reference to real examples, for example scenario 2, which is motivated by the example of type 1 diabetes, and principles, for example that main effects are generally larger than interaction terms.

### 1. Unequal variants: valid instruments with different-sized effects

In practice, it may be that some genetic variants have stronger associations with the risk factor than others. To model this, we draw the genetic effect sizes 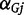 for each genetic variant *j* from independent normal distributions with mean 0.06 and standard deviation 0.018; so nearly all of the genetic effects sizes are between 0.02 and 0.12. In addition to an unweighted standard allele score where each risk-increasing allele contributed the same value to the allele score, we construct weighted allele scores (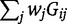). The weights (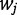) are determined in six ways: internally from the same data used in the analysis using naive and two cross-validation approaches, externally from first a small and then a large independent source, and from the coefficients in the generating model.

In the first case, taking the weights from the data under analysis (internal weights), the estimate from a weighted allele score method is the same as that obtained from a 2SLS method with a separate coefficient for each variant, as the weights are the same as the coefficients from the first-stage regression in the 2SLS analysis. In the second case, using 2-fold cross-validation, the sample is divided randomly into two equal halves. Two sets of weights are estimated in the separate halves of the data (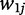, 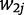 estimated in the first and second halves of the data, respectively). The weights estimated in the first half of the data are taken to construct a score for participants in the second half (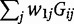 for participants *i* = 1501 , … , 3000 in the second half of the data), and vice versa (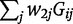 for participants *i* = 1 , … , 1500 in the first half of the data). In this way the correlation between the weights and the data under analysis is removed. In the third case, a 10-fold cross-validation approach is used, so that 10 estimated sets of weights are calculated, each using 90% of the available data, rather than 50%. In the fourth and fifth cases, weights are generated by sampling from a normal distribution around the true weight with a standard deviation of 0.04 and of 0.01. This represents uncertainty in the estimation of weights taken from the regression of the risk factor on the variants in an external data source of approximately the same size as the original dataset (3000 participants, imprecise weights) and of 16 times the size of the original dataset (48 000 participants, precise weights). In the final case, the coefficients from the generating model are the true weights.

### 2. Main and secondary variants: valid instruments with a few large and many small effects

In some practical examples, a small number of main variants have large effects (here, two) and other secondary variants may have smaller effects, a model called a ‘major-gene/polygene model’ by Pierce *et al.*^14^ We additionally consider a composite approach using 2SLS, estimating separate coefficients for the main variants and including others in an unweighted allele score. This is compared with the weighted and unweighted allele score methods discussed above and the 2SLS and LIML methods. In the generating model, the effect size for the two main variants is set at five times the size of the effect of the secondary variants. We set 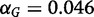 for the secondary variants and 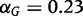 for the main variants so that the proportion of variation in the risk factor explained by the allele score (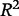) is maintained at 1.9%.

### 3. Selected variants: instruments chosen due to strength of association in the data under analysis

In practice, it may be that the investigator is uncertain if each of the alleles is truly associated with the risk factor in the population of interest and decides to include in an allele score only the variants which show the strongest association with the risk factor. To illustrate this approach, variants were ranked according to their strength of association with the risk factor and selected using two criteria: a fixed number of variants (5, 10), and a threshold *P*-value (0.05, 0.01). Estimates were obtained using an unweighted allele including only the selected variants.

### 4. Non-linear genetic effects: valid instruments with non-linear effects

In practice, it may be that some genetic variants do not have linear (that is additive or per allele) effects on the risk factor. We modify the data-generating model (1) by replacing the first line with:

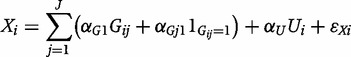

where 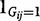 is an indicator function, taking the value one when the subscripted condition is satisfied and zero otherwise. We set 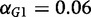 and draw the effects 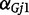 from a normal distribution with mean 0 and standard deviation 0.036. For heterozygotes (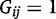), nearly all values of 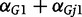 are in the range −0.02 to 0.14; 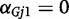 corresponds to a recessive genetic model (heterozygotes grouped with major homozygotes), and 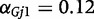 to a dominant model (heterozygotes grouped with minor homozygotes).

### 5. Interactions between genetic variants: valid instruments with genetic interactions

In practice, it may be that there are statistical interactions between the genetic variants. These are often called gene–gene interactions, though are more properly thought of as variant–variant interactions.^32^

We modify the data-generating model (1) by replacing the first line with:

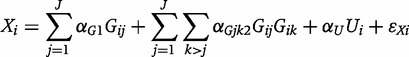



We set 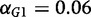 and draw the effects 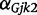 from a mixture distribution taking the value zero with probability 0.9 and a random value from a normal distribution with mean 0 and standard deviation 0.036 with probability 0.1. With 25 genetic variants, in each simulated dataset there will be an average of 30 interactions between genetic variants out of the 300 pairs of variants; these include a range of interactions from strongly negative (e.g. 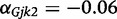) to strongly positive (e.g. 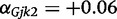).

### 6. Interactions between a genetic variant and a covariate: valid common instruments with environmental interactions

In practice, it may be that there are statistical interactions between a genetic variant and a covariate which is not a confounder. These are often called gene–environment interactions, though are more properly thought of as examples of effect modification.

We modify the data-generating model (1) by replacing it with:

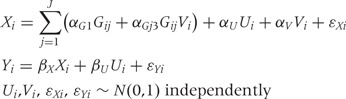

The variable *V* is introduced as a covariate affecting the risk factor but not the outcome, so that *V* is not a confounder but an effect-modifier. To ensure that the model is similar to those considered previously in terms of instrument strength, we let 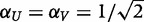. We set 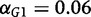 and draw the modifying effects 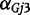 from a mixture distribution taking the value zero with probability 0.5 and a random value from a normal distribution with mean 0 and standard deviation 0.036 with probability 0.5. With 25 genetic variants, in each simulated dataset there will be an average of 12.5 interactions between a genetic variant and the covariate.

In each of scenarios 4, 5 and 6, an unweighted allele score (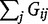) is used as an instrumental variable. This score does not account for the non-linearity and interaction terms, and is therefore misspecified for the true association of the variants with the risk factor.

### 7. Association between a genetic variant and a confounder: invalid instruments

In practice, it may be that some of the genetic variants are not specifically associated with the risk factor of interest, but instead with another variable which is a confounder in the association between the risk factor and outcome. Although they will be correlated with the factor of interest, this will be due to the effect of the confounder rather than a direct effect of the variant on the risk factor.

Unlike the previous departures from the data-generating model, which represent misspecification of the analysis model, in this case the departure is a violation of the instrumental variable assumptions. If the confounder is unmeasured, it will be impossible empirically to distinguish between this scenario and the initial scenario.

We here consider pleiotropic associations of variants with the unmeasured confounder *U*. We modify the data-generating model (1) by replacing the first line with:

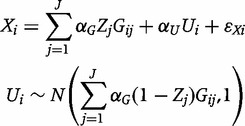

The 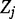 are dummy variables taking the value one if the genetic variant *j* is directly associated with the risk factor *X* (a valid instrument) and zero if the variant is associated with the confounder *U* (an invalid instrument). The strength of association between the variant and either *X* or *U* is constrained to be the same. We draw the 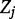 randomly, taking the probability of the instrument being valid as 0.9, 0.7 and 0.5.

## Results

We initially present the results from the initial analysis to demonstrate the performance of the methods in an idealized setting, before giving those from the various additional situations considered.

### Results of initial analysis

Table 1 displays results from each method: the median estimate across simulations, interquartile range (IQR) of estimates, coverage and power. The median estimate is given rather than the mean as the distribution of estimates has several extreme values. With the allele score and LIML methods, the theoretical mean estimate is undefined.^33^ For each number of variants and set of parameter values, both in the initial analysis and in each of the additional scenarios, the Monte Carlo standard error (the expected variation from the true value due to the limited number of simulations) of the median estimate is approximately 0.004, and of the coverage is 0.7%.

**Table 1:** Instrumental variable estimates for genetic variants with equal-sized effects from allele score analysis and multivariable analyses using two-stage least squares (2SLS) and limited information maximum likelihood (LIML) methods: mean F statistic from regression of risk factor on the instrument (F stat), median estimate across simulations, interquartile range (IQR) of estimates, coverage (Cov %) and power (%)

		**Null effect ** 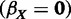			**Small effect ** 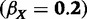				**Moderate effect ** 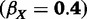			
	**F stat**	**Median**	**IQR**	**Cov %**	**Median**	**IQR**	**Cov %**	**Power**	**Median**	**IQR**	**Cov %**	**Power**
Data-generating model with 9 genetic variants												
Unweighted score	58.0	0.00	0.19	95.0	0.20	0.19	94.5	35.6	0.40	0.17	96.7	79.7
All variants (2SLS)	7.3	0.06	0.17	90.8	0.26	0.17	89.1	55.8	0.47	0.16	89.7	91.8
All variants (LIML)	7.3	0.00	0.20	95.0	0.20	0.20	94.0	39.5	0.41	0.19	95.7	77.8
Data-generating model with 25 genetic variants												
Unweighted score	58.6	0.00	0.18	96.9	0.20	0.19	95.2	36.3	0.40	0.18	96.6	77.5
All variants (2SLS)	3.3	0.15	0.14	69.2	0.35	0.14	68.8	86.9	0.55	0.14	67.9	99.1
All variants (LIML)	3.3	0.01	0.21	92.6	0.20	0.20	92.4	36.2	0.40	0.22	93.5	72.8
Data-generating model with 100 genetic variants												
Unweighted score	57.4	−0.01	0.18	95.4	0.20	0.18	95.7	35.7	0.40	0.17	95.2	77.0
All variants (2SLS)	1.6	0.32	0.10	1.3	0.52	0.09	1.4	100.0	0.72	0.09	0.9	100.0
All variants (LIML)	1.6	−0.01	0.30	79.2	0.21	0.30	80.5	42.6	0.41	0.27	82.1	70.2

We see that the estimates using 2SLS are biased throughout and coverage is less than the nominal 95% level. Bias acts in the direction of confounding, and is especially serious with large numbers of genetic variants of smaller effect size. Although the power reaches 100% in some cases, this is meaningless when the coverage is below the nominal level and is due to the large bias. The LIML estimates show good performance with bias compatible with zero, but coverage levels decrease as the number of variants increases. This is a known problem, and coverage can be improved by using a correction to the method due to Bekker.^34^ However, Bekker standard errors are not available in the *ivreg2* command in Stata, and so the correction has not been implemented here as it is likely that practitioners would use the default option. The median estimates using the allele score are unbiased with correct coverage levels throughout. The precision of the allele score method is greater (i.e. has a lower IQR) than LIML, but the power is similar (where the coverage of each method is close to the correct 95%). This is expected for variants with equal-sized effects as, in this case with a correctly specified allele score, no information is lost by converting the multivariate data on genetic variants to a univariate unweighted allele score.

In summary, when correctly specified, allele scores allow valid estimation of causal effects using large numbers of genetic variants where conventional methods (2SLS, LIML) suffer from problems of bias and/or reduced coverage (overly narrow confidence intervals). The LIML approach is a reasonable alternative (with less than 10 variants) or a sensitivity analysis (with large numbers of variants) as it is approximately unbiased even with large numbers of instruments.

#### Results of additional scenarios

Table 2 gives results for each of the seven additional scenarios described above for data-generating models with 25 genetic variants. Results for 9 and 100 genetic variants are given in the Appendix (see Supplementary data available at *IJE* online). In each case, we present the median estimates across simulations, and the coverage; the IQR of estimates for a null effect and power for a non-null effect are also shown.

**Table 2:** Instrumental variable estimates in a range of scenarios from allele score analysis and multivariable analyses using two-stage least squares (2SLS) and limited information maximum likelihood (LIML) methods in data-generating model with 25 genetic variants: mean F statistic from regression of risk factor on the instrument (F stat), median estimate across simulations, interquartile range (IQR) of estimates, coverage (Cov %) and power (%)

		**Null effect (** 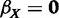 )			**Small effect** 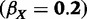			**Moderate effect** 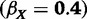		
	**F stat**	**Median**	**IQR**	**Cov %**	**Median**	**Cov %**	**Power**	**Median**	**Cov %**	**Power**
1. Unequal effects										
Unweighted score	58.5	0.00	0.18	96.7	0.20	95.3	36.3	0.40	96.7	76.7
Internal weights (2SLS)^a^	89.2	0.14	0.13	71.7	0.34	70.3	87.6	0.54	68.9	99.3
Cross-validated weights (2-fold)	32.2	0.00	0.26	96.1	0.20	94.6	25.4	0.40	95.9	56.5
Cross-validated weights (10-fold)	43.1	-0.01	0.22	95.8	0.20	94.8	26.8	0.40	96.5	62.5
External weights (imprecise)	46.2	0.00	0.20	96.5	0.20	94.9	32.3	0.40	97.2	68.3
External weights (precise)	62.4	0.00	0.17	95.7	0.20	94.9	38.9	0.40	97.1	80.2
True weights	64.0	0.00	0.17	96.3	0.21	94.5	38.9	0.40	96.6	80.3
LIML	3.5	0.00	0.20	92.4	0.20	92.2	38.8	0.40	94.2	77.2
2. Main and secondary effects										
Unweighted score	59.2	0.00	0.18	96.9	0.20	95.2	36.8	0.40	96.6	78.3
Internal weights (2SLS)	124.5	0.10	0.12	76.6	0.30	90.2	78.2	0.50	77.3	99.8
Cross-validated weights (2-fold)	58.8	0.00	0.20	96.1	0.19	95.1	35.7	0.41	95.1	75.3
Cross-validated weights (10-fold)	75.0	0.00	0.17	95.8	0.19	94.9	40.1	0.40	95.4	82.8
External weights (imprecise)	79.2	0.00	0.15	95.3	0.20	94.7	42.0	0.40	95.9	86.7
External weights (precise)	97.6	0.00	0.14	95.9	0.20	95.1	48.1	0.40	95.9	92.8
True weights	99.2	0.00	0.14	95.7	0.20	94.9	49.4	0.40	95.7	92.9
Composite approach	33.8	0.01	0.14	95.2	0.21	94.9	53.4	0.41	95.1	94.0
LIML	4.9	0.00	0.16	93.3	0.20	93.6	49.7	0.40	92.6	91.6
3. Selected variants										
Top 5 variants	40.9	0.23	0.12	62.4	0.43	60.7	81.4	0.64	58.5	97.5
Top 10 variants	59.8	0.19	0.10	64.5	0.39	63.4	85.9	0.59	63.2	98.6
Variants with 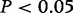	54.9	0.21	0.10	61.8	0.41	58.6	86.1	0.62	57.5	98.1
Variants with 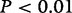	34.8	0.26	0.10	59.1	0.45	62.8	73.2	0.67	58.7	90.5
4. Non-linear effects										
Unweighted score	58.5	0.00	0.18	96.8	0.20	95.2	36.3	0.40	96.7	76.8
5. Interactions between variants										
Unweighted score	59.5	0.00	0.18	96.6	0.20	95.5	37.3	0.40	96.6	77.5
6. Interactions between a variant and covariate										
Unweighted score	44.8	0.00	0.18	96.9	0.20	95.5	36.2	0.40	96.8	77.1
7. Invalid variants										
90% valid variants	58.6	0.10	0.19	83.3	0.30	82.7	61.0	0.50	84.2	90.1
70% valid variants	58.6	0.30	0.21	35.8	0.50	35.9	91.9	0.70	35.7	98.2
50% valid variants	58.6	0.49	0.20	6.2	0.70	5.8	99.0	0.89	4.9	100.0

^a^The point estimate of a weighted allele score with internally-derived weights (weights derived from the data under analysis) is the same as that from the 2SLS method with a separate coefficient for each variant.

We see as follows:

##### Scenarios 1–2

For variants with different sizes of effect, the use of true weights rather than an unweighted allele score gave some improvement in power. When the alleles had similar sizes of effect (scenario 1), the gain in power was generally only 3–4%, whereas when the alleles had considerably different sizes of effect (scenario 2), the gain was 12–15%. Results using an unweighted allele score were unbiased even though the model was misspecified. Naive use of weights derived from the same data under analysis resulted in severe bias. Use of precisely estimated externally derived weights was as efficient as use of the true weights, although power was reduced when the weights were less precisely estimated even, in some cases, to below that of the unweighted score. Estimates using weights from a cross-validation approach were unbiased, with power in the 10-fold cross-validation analysis slightly below that with the imprecisely measured external weights, and in the 2-fold cross-validation analysis lower still due to the weights being estimated in a smaller sample. In scenario 2, the composite method results using three instrumental variables indicate a small amount of bias consistent with weak instrument bias. Although nominal coverage levels appear to be maintained, the apparent power is slightly greater than when the true weights are used, possibly due to the slight upward bias in estimates. Composite approaches should only be used therefore when there are variants with substantially different magnitudes of association, and where the composite instrument is reasonably strong.

##### Scenario 3

The use of variants chosen according to their strength of association with the risk factor in the data under analysis gave seriously biased estimates, with bias in the direction of the confounded association. The bias is a result of the so-called ‘winner's curse’, whereby the estimate of the lead variant's association with the risk factor is likely to be overestimated because of chance correlation with confounders, leading to bias in the estimate of the causal effect.

##### Scenarios 4–6

None of the ways of misspecifying the analysis model considered (non-linear genetic effects, variant–variant and variant–environment interactions) affected the bias, coverage or power of estimates using the unweighted allele score.

##### Scenario 7

The use of invalid genetic variants in an allele score severely biased estimates of causal effects, even when 90% of the variants in the score were valid instruments.

To summarize, the use of an allele score did not seem to be sensitive to implicit parametric assumptions made by the procedure, such as the linearity of the genetic associations. However, estimates are sensitive to how the score is constructed, both how the variants included in the score are chosen and how the weights in a weighted score are determined.

## Discussion

The overall conclusion from this simulation study is that unweighted allele scores can be used as instruments in Mendelian randomization if each of the variants used in constructing the allele score satisfies the assumptions of an instrumental variable. We consider an estimator to be valid if it is consistent for the parameter of interest, the finite-sample bias is not large and the nominal coverage of confidence intervals is maintained. The validity of the unweighted allele score did not appear to be adversely affected by misspecifications of the genetic model, at least in the range of simulation examples considered, such as the assumption of equal effect sizes for variants, non-linear genetic effects, or effect modification by variant–variant or variant–environment interactions. This is important because, in practice, the true genetic model is unknown.

When an allele score is proposed for use in a Mendelian randomization analysis, researchers should make clear precisely how the decisions leading to the construction of the score were made. If variants have different sizes of effect on the risk factor, then precision can be gained by using a weighted allele score, although the use of an unweighted score gave reasonable estimates in the examples considered. If variants have considerably different sizes of effect, then a weighted allele score would be thought to be advisable, although the weights should not be generated naively from the data under analysis.^14^ If the weights are imprecisely measured, then estimates remain unbiased, although gains in power are somewhat reduced. In practice, if the only source of information on the weights is the data under analysis, then a cross-validation approach can be undertaken. We would recommend a 10-fold cross-validation approach, so that in each case the weights are calculated according to 90% of the data, and 10 sets of weights are required. A jackknife (or *N*-fold cross-validation, where *N* is the sample size) approach may give even greater precision.^35^ In a jackknife approach, a set of weights is calculated for each participant using data on all of the other participants. This was not attempted in this paper because of the computational intensity of the method.

The use of an allele score enables reliable instrumental variable analysis with much larger numbers of genetic variants than conventional methods (2SLS, LIML) can handle. Although LIML performed reasonably well in terms of bias, the coverage of the LIML estimate was below nominal levels with large numbers of variants. For variants with different sizes of effect on the risk factor, LIML gave improved power over an unweighted score method, but did not dominate a weighted allele score method in terms of precision. Estimates from the 2SLS method showed bias and poor coverage throughout, a manifestation of the problems of weak instrument bias.

In order to make comparisons across simulations with different numbers of variants, we have assumed that the effect size is smaller when there are more variants in the allele score. In practice, there is no trade-off that the effect size decreases as the number of instruments increases. The choice as to how many (and which) variants to include in an allele score should be a question addressed using scientific knowledge rather than statistical testing. Unless variants are very highly correlated, all variants which can be reasonably assumed to be valid instruments should be included in a Mendelian randomization analysis to improve the precision of the causal estimate.^15^

The use of large numbers of genetic variants associated with a risk factor has been proposed in Mendelian randomization, on the premise that pleiotropic effects may be expected to ‘balance out’.^36^ This is similar to expecting the effects of confounding on observational estimates of association to cancel out. The results of this paper demonstrate that the criteria for the inclusion of a genetic variant in an allele score should be just as stringent as those for any other Mendelian randomization analysis.

### Comparison with previous work

Previous work on the use of multiple genetic variants in the context of Mendelian randomization has demonstrated that using an allele score results in increases in power compared with using single genetic variants, with slight reductions in power compared with using multiple variants, but better bias properties.^14^^,^^15^ This paper confirms these findings and further reveals the problem of poor coverage with large numbers of variants. The additional contributions of this paper are: the comparison of internally weighted, externally weighted and unweighted allele scores; the use of cross-validation to obtain internally-derived weights; the comparison of external weights with different precisions; the investigation of data-driven selections of variants to use in allele scores; the inclusion of LIML as well as 2SLS; and addressing the robustness of estimates using allele scores to misspecification of the score. A key novel finding of this paper affecting the use of allele scores in practice is that the procedure used for constructing an allele score, or for deriving weights for a weighted score, has a considerable impact on the bias of estimates.

### Limitations of this paper

Although the simulations have covered a range of different scenarios, the conclusions are limited by the reliance on simulated rather than theoretical results. Different simulation parameters could be investigated in further investigations. In response to concerns from a reviewer, simulations were repeated with a 10-fold larger sample size; overall findings were unchanged (Table A3, available as Supplementary Data at *IJE* online). Further departures from the analysis model in the data-generating model could be considered. For example, we have here considered genetic interactions on a linear scale; interactions could be considered on a multiplicative scale. We have limited this paper to the case of a continuous outcome. Although the outcome in Mendelian randomization is often binary, binary outcomes result in other difficulties in effect estimation.^37^^,^^38^ However, we have no reason to doubt that the general findings of this paper would be applicable to the binary case.

We have here assumed that the external weights used in calculating a weighted allele score are relevant estimates of the true weights. If the external source is from a different population, then these weights may be biased for the true weights. As the use of an unweighted score, which is known to be misspecified with variants of different strengths, did not result in bias, it is unlikely that the use of misestimated weights would lead to serious bias. However, when choosing a source to derive external weights, it is best to choose a source from a similar population with enough participants to ensure precisely estimated relevant weights. If a relevant external data source cannot be identified, then either an unweighted score (if the genetic effect sizes are similar) or a cross-validation approach (if the sample size is large or the effect sizes are diverse) would be preferred. In choosing between approaches, there is a tradeoff between an unweighted score (inefficient, but unlikely to lead to bias), a cross-validation approach (weights are relevant estimates, efficiency depends on sample size), and an external source (weights may be less relevant estimates, and may be more precisely estimated).

One assumption which we have not varied is the independence of genetic variants. If several variants are included in an allele score which are in high linkage disequilibrium (highly correlated), then it would be unnecessary to include all the variants in an allele score, especially if they all happened to be correlated with the same functional variant. This would also lead to difficulties in estimating and interpreting weights in a weighted allele score.

A disadvantage of using multiple genetic variants, and allele scores in particular, is sporadic missing data leading to reduced sample sizes for analysis.^15^ In the multiple variant setting, imputation methods have been shown to be effective in mitigating against any reduction in power due to missing data.^39^

## Supplementary Data

Supplementary data are available at *IJE* online.

## Funding

Stephen Burgess is supported by the Wellcome Trust (grant number 100114).

## Supplementary Material

Supplementary Data
