# Supplementary material for: Use of allele scores as instrumental variables for Mendelian randomization
Source: Int J Epidemiol. 2013 Aug 30;42(4):1134–44. doi: 10.1093/ije/dyt093 (PMC3780999; doi:10.1093/ije/dyt093)
Supplement: Supplementary Data [file supp_42_4_1134__index.html]

Supplementary Data 

# Use of allele scores as instrumental variables for Mendelian randomization

## Supplementary Data

files

**Files in this Data Supplement:**

- Supplementary Data - pdf file
