# Supplementary material for: Use of allele scores as instrumental variables for Mendelian randomization
Source: Int J Epidemiol. 2013 Aug 30;42(4):1134–44. doi: 10.1093/ije/dyt093 (PMC3780999; doi:10.1093/ije/dyt093)
Supplement: Supplementary Data [file supp_dyt093_ije-2012-10-1002.pdf]

Web Appendix

Additional parameters and results

A.1 Changing the number of variants

We briefly list the parameters used in the simulations in each of the seven scenarios considered with 9, 25 and 100 genetic variants.

1. **Unequal variants:** Genetic effect sizes α_{Gj} for each genetic variant j from a normal distribution with mean (0.1, 0.06, 0.03) and standard deviation (0.03, 0.018, 0.009). Independent weights are generated from a normal distribution with standard deviation of 0.04 and 0.01.

2. **Main and secondary variants:** Genetic effect sizes $\alpha_G = (0.054, 0.046, 0.0285)$ for secondary variants and $5\alpha_G = (0.27, 0.23, 0.1425)$ for two main variants.

3. **Selected variants:** The fixed numbers of variants were (2, 5, 10) and (4, 10, 20).

4. **Non-linear genetic effects:** Genetic effect sizes $\alpha_G = (0.1, 0.06, 0.03)$, effects α_{Gj1} drawn from a normal distribution with mean 0 and standard deviation (0.06, 0.036, 0.018).

5. **Interactions between genetic variants:** Genetic effect sizes $\alpha_G = (0.1, 0.06, 0.03)$, effects α_{Gjk2} drawn from a mixture distribution taking the value zero with probability (0.6, 0.9, 0.96) and a random value from a normal distribution with mean 0 and standard deviation (0.06, 0.036, 0.018) with probability (0.4, 0.1, 0.04). With (9, 25, 100) genetic variants, in each simulated dataset there will be an average of (14.4, 30, 198) interactions between genetic variants out of the (36, 300, 4950) pairs of variants.

6. **Interactions between a genetic variant and a covariate:** Genetic effect sizes $\alpha_G = (0.1, 0.06, 0.03)$, effects α_{Gj3} drawn from a mixture distribution taking the value zero with probability 0.5 and a random value from a normal distribution with mean 0 and standard deviation (0.06, 0.036, 0.018) with probability 0.5. With (9, 25, 100) genetic variants, in each simulated dataset there will be an average of (4.5, 12.5, 50) interactions between a genetic variant and the covariate.

7. **Invalid variants:** Parameters as in initial analysis.

The results with 9 and 100 genetic variants are given in Web Tables A1 and A2 respectively.

	F stat	Null effect ($\beta_X = 0$) Median	IQR	Cov %	Small effect ($\beta_X = 0.2$) Median	Cov %	Power	Moderate effect ($\beta_X = 0.4$) Median	Cov %	Power
					1. Unequal effects					
Unweighted score	58.6	0.00	0.19	95.2	0.20	94.6	36.4	0.40	96.7	78.9
Internal weights (2SLS) [1]	71.6	0.06	0.16	90.7	0.25	89.2	57.2	0.46	90.8	93.0
Cross-validated weights (2-fold)	45.7	0.00	0.22	96.8	0.19	96.2	29.9	0.40	95.7	67.3
Cross-validated weights (10-fold)	53.0	-0.01	0.19	95.9	0.20	95.1	33.3	0.40	95.9	72.2
External weights (imprecise)	55.8	0.00	0.19	96.1	0.21	94.9	35.2	0.40	95.7	77.3
External weights (precise)	62.8	0.00	0.18	95.9	0.20	95.1	39.8	0.40	96.1	80.7
True weights	63.4	0.00	0.18	95.7	0.20	95.0	40.2	0.40	96.1	80.3
LIML	7.9	0.00	0.18	95.3	0.20	93.9	40.2	0.40	95.0	79.7
					2. Main and secondary effects					
Unweighted score	59.3	0.00	0.18	95.1	0.20	94.5	37.3	0.40	96.7	81.0
Internal weights (2SLS)	113.7	0.03	0.12	92.8	0.24	92.4	69.8	0.44	93.5	98.0
Cross-validated weights (2-fold)	84.4	0.00	0.14	95.6	0.20	95.2	45.1	0.40	95.3	87.3
Cross-validated weights (10-fold)	94.1	-0.01	0.14	95.7	0.19	94.9	47.9	0.40	96.2	90.9
External weights (imprecise)	97.5	0.00	0.14	96.5	0.20	94.9	51.6	0.40	95.5	92.7
External weights (precise)	104.8	0.00	0.13	95.9	0.20	94.3	52.6	0.40	96.0	94.7
True weights	105.3	0.00	0.14	95.9	0.20	94.3	53.6	0.40	96.3	94.6
Composite approach	35.8	0.01	0.13	95.5	0.21	94.5	57.6	0.41	95.9	95.9
LIML	12.6	0.00	0.14	95.4	0.20	94.1	54.4	0.40	95.1	93.7
					3. Selected variants					
Top 2 variants	28.1	0.17	0.15	80.5	0.36	79.9	55.7	0.55	82.3	83.1
Top 4 variants	44.9	0.13	0.12	84.0	0.33	80.3	64.8	0.53	83.4	92.8
Variants with $p < 0.05$	57.9	0.08	0.10	88.5	0.28	86.9	62.8	0.48	87.9	92.8
Variants with $p < 0.01$	48.7	0.13	0.11	83.3	0.33	80.3	66.9	0.53	83.2	93.0
					4. Non-linear effects					
Unweighted score	58.3	0.00	0.19	95.1	0.19	94.5	35.9	0.40	96.8	79.5
					5. Interactions between variants					
Unweighted score	69.2	0.00	0.19	97.2	0.19	95.2	37.1	0.41	94.4	77.7
					6. Interactions between a variant and covariate					
Unweighted score	46.0	0.00	0.19	95.3	0.20	94.7	35.7	0.40	96.9	79.7
					7. Invalid variants					
90% valid variants	58.0	0.09	0.20	80.2	0.29	77.9	58.4	0.49	80.2	89.9
70% valid variants	58.0	0.30	0.26	36.9	0.51	37.2	87.9	0.71	35.1	97.0
50% valid variants	58.0	0.49	0.27	10.5	0.69	11.4	97.5	0.89	9.4	99.5

Web Table A1: Instrumental variable estimates in a range of scenarios from allele score analysis and multivariable analyses using two-stage least squares (2SLS) and limited information maximum likelihood (LIML) methods in data-generating model with 9 genetic variants: mean F statistic from regression of risk factor on the instrument (F stat), median estimate across simulations, interquartile range (IQR) of estimates, coverage (Cov %) and power (%)

[1] The point estimate of a weighted allele score with internally-derived weights (weights derived from the data under analysis) is the same as that from the 2SLS method with a separate coefficient for each variant.

	F stat	Null effect ($\beta_X = 0$) Median	IQR	Cov %	Small effect ($\beta_X = 0.2$) Median	Cov %	Power	Moderate effect ($\beta_X = 0.4$) Median	Cov %	Power
	1. Unequal effects									
Unweighted score	57.3	-0.01	0.19	95.4	0.20	95.7	35.5	0.40	95.3	76.3
Internal weights (2SLS) [1]	166.8	0.31	0.10	2.1	0.51	1.8	100.0	0.71	1.0	100.0
Cross-validated weights (2-fold)	13.0	0.01	0.48	94.8	0.21	95.0	19.9	0.40	95.1	33.9
Cross-validated weights (10-fold)	21.5	-0.01	0.36	94.3	0.21	94.0	23.4	0.41	94.0	45.2
External weights (imprecise)	24.4	-0.01	0.28	95.4	0.22	95.6	23.4	0.40	96.3	48.3
External weights (precise)	56.7	-0.01	0.20	95.5	0.20	95.6	36.6	0.39	95.3	75.2
True weights	62.4	-0.01	0.18	95.0	0.20	95.8	38.4	0.40	95.5	78.7
LIML	1.6	0.00	0.29	79.9	0.21	80.0	43.9	0.40	81.9	71.9
	2. Main and secondary effects									
Unweighted score	60.0	-0.01	0.18	95.5	0.20	95.7	37.0	0.40	95.3	78.5
Internal weights (2SLS)	181.7	0.29	0.10	2.3	0.49	2.3	100.0	0.68	2.0	100.0
Cross-validated weights (2-fold)	17.8	0.01	0.39	95.4	0.21	95.2	22.1	0.40	94.9	40.4
Cross-validated weights (10-fold)	29.4	0.00	0.29	95.1	0.21	93.9	27.2	0.40	94.8	54.2
External weights (imprecise)	33.2	0.00	0.24	95.1	0.22	95.8	26.9	0.39	96.9	58.6
External weights (precise)	70.6	0.00	0.16	95.0	0.21	96.4	42.5	0.39	94.8	82.1
True weights	76.5	-0.01	0.15	94.9	0.20	95.8	45.9	0.40	95.6	85.3
Composite approach	26.2	0.01	0.15	94.4	0.22	95.5	50.6	0.41	94.3	89.0
LIML	1.8	0.00	0.16	82.6	0.21	83.0	47.5	0.41	86.1	78.7
	3. Selected variants									
Top 20 variants	79.3	0.30	0.09	19.7	0.51	19.0	97.9	0.71	19.7	99.8
Top 40 variants	91.0	0.24	0.09	32.6	0.45	31.1	96.9	0.64	30.7	99.7
Variants with $p < 0.05$	62.4	0.32	0.10	24.5	0.32	19.8	96.6	0.73	20.5	99.0
Variants with $p < 0.01$	30.0	0.37	0.17	40.0	0.37	39.3	80.2	0.76	41.3	90.1
	4. Non-linear effects									
Unweighted score	57.2	-0.01	0.19	95.4	0.20	95.8	35.8	0.40	95.2	76.5
	5. Interactions between variants									
Unweighted score	56.9	-0.01	0.18	95.5	0.20	95.6	34.5	0.40	95.2	75.8
	6. Interactions between a variant and covariate									
Unweighted score	44.6	-0.01	0.19	96.6	0.20	96.2	35.1	0.40	95.6	77.4
	7. Invalid variants									
90% valid variants	57.4	0.09	0.18	83.4	0.30	84.6	62.6	0.49	84.2	90.2
70% valid variants	57.4	0.30	0.18	34.4	0.50	31.3	95.0	0.70	33.2	98.8
50% valid variants	57.4	0.49	0.18	3.4	0.70	3.0	99.6	0.90	3.7	100.0

Web Table A2: Instrumental variable estimates in a range of scenarios from allele score analysis and multivariable analyses using two-stage least squares (2SLS) and limited information maximum likelihood (LIML) methods in data-generating model with 100 genetic variants: mean F statistic from regression of risk factor on the instrument (F stat), median estimate across simulations, interquartile range (IQR) of estimates, coverage (Cov %) and power (%)

[1] The point estimate of a weighted allele score with internally-derived weights (weights derived from the data under analysis) is the same as that from the 2SLS method with a separate coefficient for each variant.

A.2 Changing the sample size

In response to concern from a reviewer that the findings of this paper may only apply in small sample settings, we repeated the simulations for 25 variants with a sample size of 30 000. Results are given in Table A3 and show no substantial differences from those previously presented with a sample of size 3000, with the exception of Scenario 3, where imposing a p-value threshold for variants no longer resulted in substantial bias. This is because, with the increased sample size, all 25 variants ($p < 0.05$) or at least 24 of the 25 variants ($p < 0.01$) were chosen in over 90% of simulated datasets. While the findings of this paper are limited by their reliance on the results of simulation analyses, we have no reason to suspect that the paper's recommendations are sensitive to the sample size.

		Null effect ($\beta_X = 0$)			Small effect ($\beta_X = 0.2$)			Moderate effect ($\beta_X = 0.4$)		
	F stat	Median	IQR	Cov %	Median	Cov %	Power	Median	Cov %	Power
Unweighted score	568.6	0.00	0.06	95.7	0.20	95.5	99.3	0.40	94.4	100
2SLS	23.7	0.02	0.06	91.7	0.22	91.8	99.8	0.42	91.1	100
LIML	23.7	0.00	0.06	95.2	0.20	94.7	99.0	0.40	94.5	100
	1. Unequal effects									
Unweighted score	545.3	0.00	0.06	95.7	0.20	95.5	99.1	0.40	94.4	100.0
Internal weights (2SLS) [1]	624.8	0.02	0.05	92.5	0.22	91.3	99.9	0.42	91.2	100.0
Cross-validated weights (2-fold)	536.3	0.00	0.06	95.7	0.20	95.0	98.5	0.40	94.3	100.0
Cross-validated weights (10-fold)	570.5	0.01	0.06	95.7	0.20	95.3	98.9	0.40	94.4	100.0
External weights (imprecise)	420.1	0.00	0.06	94.0	0.20	95.1	95.8	0.40	93.5	100.0
External weights (precise)	587.0	0.00	0.06	95.6	0.20	95.3	99.6	0.40	94.4	100.0
True weights	600.4	0.00	0.05	96.1	0.20	94.8	99.7	0.40	94.3	100.0
LIML	25.0	0.00	0.06	95.5	0.20	95.1	99.3	0.40	93.9	100.0
	2. Main and secondary effects									
Unweighted score	581.1	0.00	0.06	95.7	0.20	95.5	99.4	0.40	94.4	100.0
Internal weights (2SLS)	974.5	0.01	0.04	92.9	0.21	93.0	100.0	0.41	92.5	100.0
Cross-validated weights (2-fold)	911.0	0.00	0.05	94.8	0.20	95.4	100.0	0.40	95.7	100.0
Cross-validated weights (10-fold)	944.5	0.00	0.05	95.0	0.20	95.7	99.8	0.40	95.5	100.0
External weights (imprecise)	767.4	0.00	0.05	95.0	0.20	95.8	99.9	0.40	94.6	100.0
External weights (precise)	958.3	0.00	0.05	95.3	0.20	95.9	99.9	0.40	95.6	100.0
True weights	1000.5	0.00	0.05	95.5	0.20	95.9	100.0	0.40	95.8	100.0
Composite approach	325.5	0.00	0.05	95.4	0.20	95.5	100.0	0.40	95.4	100.0
LIML	40.0	0.00	0.05	94.8	0.20	95.8	100.0	0.40	95.7	100.0
	3. Selected variants									
Top 5 variants	184.7	0.11	0.08	66.3	0.31	63.5	98.6	0.51	66.0	100.0
Top 10 variants	321.8	0.08	0.07	66.9	0.28	63.6	99.7	0.48	67.7	100.0
Variants with $p < 0.05$	564.4	0.00	0.05	95.0	0.20	95.2	99.5	0.40	94.5	100.0
Variants with $p < 0.01$	564.2	0.00	0.06	95.2	0.21	95.0	99.5	0.41	94.4	100.0
	4. Non-linear effects									
Unweighted score	571.7	0.00	0.06	95.7	0.20	95.5	99.2	0.40	94.4	100.0
	5. Interactions between variants									
Unweighted score	316.0	0.00	0.05	95.8	0.20	95.4	92.3	0.40	94.3	100.0
	6. Interactions between a variant and covariate									
Unweighted score	433.1	0.00	0.04	95.7	0.20	95.5	99.3	0.40	94.5	100.0
	7. Invalid variants									
90% valid variants	570.0	0.09	0.06	40.6	0.30	39.3	99.8	0.50	39.1	100.0
70% valid variants	570.0	0.30	0.09	0.9	0.50	1.0	100.0	0.70	0.7	100.0
50% valid variants	570.0	0.50	0.09	0.0	0.70	0.0	100.0	0.90	0.0	100.0

Web Table A3: Instrumental variable estimates in a range of scenarios from allele score analysis and multivariable analyses using two-stage least squares (2SLS) and limited information maximum likelihood (LIML) methods in data-generating model with 25 genetic variants and large sample size (30 000 individuals): mean F statistic from regression of risk factor on the instrument (F stat), median estimate across simulations, interquartile range (IQR) of estimates, coverage (Cov %) and power (%)

[1] The point estimate of a weighted allele score with internally-derived weights (weights derived from the data under analysis) is the same as that from the 2SLS method with a separate coefficient for each variant.
